# Impact of the Physical Modification of Starch (*Oxalis tuberosa*) in a Low-Fat Snack by Hot Air Frying, a Sustainable Process

**DOI:** 10.3390/foods14162909

**Published:** 2025-08-21

**Authors:** Nayeli Anayansi Loyo-Trujillo, María Remedios Mendoza-López, Rosa Isela Guzmán-Gerónimo, Rosario Galvan-Martínez, Francisco Erik González-Jiménez, Josué Antonio del Ángel-Zumaya, Audry Peredo-Lovillo, Juan Vicente Méndez-Méndez

**Affiliations:** 1 Centro de Investigación y Desarrollo de Alimentos, Universidad Veracruzana, Xalapa 91190, Veracruz, Mexico; anayansip.v.89@gmail.com; 2 Instituto de Química Aplicada, Universidad Veracruzana, Xalapa 91190, Veracruz, Mexico; 3 Instituto de Ciencias Básicas, Universidad Veracruzana, Xalapa 91190, Veracruz, Mexico; rgalvan@uv.mx; 4 Facultad de Ciencias Químicas, Universidad Veracruzana, Orizaba 94340, Veracruz, Mexico; franciscogonzalez02@uv.mx (F.E.G.-J.); jodelangel@uv.mx (J.A.d.Á.-Z.); auperedo@uv.mx (A.P.-L.); 5 Centro de Nanociencias y Micro y Nanotecnologías, Instituto Politécnico Nacional, Ciudad de Mexico 07738, Mexico; jmendezm@ipn.mx

**Keywords:** *Oxalis tuberosa* starch, microwaves, liquid nitrogen, hot air frying, vegan snack, food quality, food safety

## Abstract

Currently, there is an increasing demand for plant-based and low-fat snacks. Non-conventional starch and grains are alternative ingredients. Environmentally friendly processing, such as liquid nitrogen and microwaves, can be used to obtain modified starch, as well as hot air frying to cook snacks. The aim of this work was to evaluate the impact of eco-friendly physical modification of starch from *Oxalis tuberosa* in a low-fat snack processed by hot air frying. First, native starch (NS) was treated with liquid nitrogen (LNS) and liquid nitrogen/microwaves (LNMS), and the amylose/amylopectin content and functional properties were determined. The snacks were formulated with NS or modified starches, amaranth flour, quinoa flour, corn, onion powder, salt, and water; the ingredients were mixed and placed in an electric pasta maker and cooked by hot air frying. The hardness, hedonic test, colorimetric parameters, acrylamide, proximal composition, and fatty acid profile were analyzed. All starches showed similar values of amylose and amylopectin content. LNMS starch had the lowest water solubility index as compared to NS and LNS. The snacks with the starch modified with liquid nitrogen showed the highest values of hardness as well as the highest score for the texture from a hedonic test. The snacks with modified starches showed a lower browning index than the snack formulated with NS. Acrylamide was not detected in any snacks. The lipid value of the snacks with modified starch was 1.9–2.70 g/100 g of sample, providing ω-9, ω-6, and ω-3 fatty acids. All snacks contained 7.7 g of protein/100 g of sample. These low-fat and plant-based snacks are a healthy option made by environmentally friendly technologies.

## 1. Introduction

Snack can be defined as a small meal between meals, including a wide range of food products [1]. Currently, more consumers are frequently replacing at least one meal with snacks due to their accessibility and demand for lower-oil and gluten-free options, with a strong focus on plant-based and protein-enriched snacks processed by eco-friendly technologies.

Several factors should be considered to provide a desirable health product with the proper sustainable technology. A wide variety of grains can be incorporated into plant-based snacks, such as quinoa and amaranth, which are pseudo-cereal sources of high-quality protein as well as unsaturated fatty acids. A previous study indicates that snacks formulated with quinoa or amaranth showed a higher protein content as well as high contents of unsaturated fatty acids [2].

In addition, starch is a carbohydrate widely used in the food products formulation. A conventional starch source in snack formulation is potato (*Solanum tube tuberosum rosum* L.). However, harmful chemical compounds, such as acrylamide, may be produced, mainly by the Maillard reaction, during the processing of potato snacks [3]. Non-conventional starch of *Oxalis tuberosa* represents an alternative ingredient for the snack industry. In order to improve its applications even further, native starch is frequently modified.

Chemically modified starches are widely used ingredients in the industry; however, chemical treatment of the starch can have a negative impact on the environment. Easy and environmentally friendly technologies for the physical modification of starch are available [4], such as microwaves and freezing with liquid nitrogen [5,6,7].

Fast freezing with liquid nitrogen produces a quick freezing and can minimize structural damage to the starch as well as improve its physicochemical properties [8,9,10,11]. In addition, it has been reported that liquid nitrogen maintains the protein content, which has a nutritional benefit [12]. Also, the physical modification of starch has an influence on the texture, an important parameter for the sensory quality of the snacks. A previous study indicated that freezing with liquid nitrogen improved the texture of Liangpi, a traditional Chinese snack [13].

Microwaves are electromagnetic waves that interact with water molecules bound to starch, resulting in rapid heating, modifying functional properties such as solubility and swelling power. Starch modified by microwaves has a potential application in the elaboration of products that require low expansion, such as cookies and potato chips, keeping the desired shape [14].

The goal of eco-friendly modified starches is to have a positive impact on food quality. Previous work has reported an improvement in potato starch when modified by microwaves, resulting in a better texture of fresh noodles [15]. Another positive result was observed in puffed yellow pea snacks processed by nitrogen-gas-assisted extrusion [16]. Also, starch treated by microwaves produced an improvement in the browning index of food products [17]. Besides the type of modified starch used to formulate starchy foods, the processing technology also plays a crucial role in the nutritional, safety, and sensory characteristics of the final product. Environmentally friendly technologies, such as hot air frying, are an alternative to the use of oil in the processing of snacks. In addition, low-fat and a low content of acrylamide can be obtained in foods via hot air frying [18,19]. So, it is important to explore sustainable methods of starch modification as well as processing technologies in food products. The aim of this work was to evaluate the impact of starch from *Oxalis tuberosa* modified with liquid nitrogen and liquid nitrogen/microwaves on the quality of a low-fat, plant-based snack processed by hot air frying.

## 2. Materials and Methods

### 2.1. Materials

Mexican *Oxalis tuberosa* tubers were collected in November 2023 in Agua Escondida in the State of Veracruz, Mexico (latitude 18°58′43″ N 97° 12′ W, at 2760 masl). Amaranth flour, quinoa flour, onion powder, and salt were bought from a local market in Xalapa, Veracruz.

### 2.2. Physical Modification of the Native Starch

The native starch was obtained according to the Nuñez-Breton et al. [20]. The tubers were blended in water and filtered. The filtrate was left to rest for 3 h at 25 °C for starch precipitation. This procedure was repeated two more times. The precipitate was dried in a convection oven (Ecoshell, Mod. 9025 E, Mexico, 40 °C for 24 h).

The physical modification of starch was performed using liquid nitrogen and liquid nitrogen/microwaves. For the modification with liquid nitrogen, dry starch (200 g) was placed in an aluminum container, and liquid nitrogen (1 L) was added; the time was 1 h. For the starch modification with liquid nitrogen/microwaves, the starch previously modified with liquid nitrogen was placed in a Petri dish (20 g) and processed in a microwave oven (Panasonic, 2450 MHz) for 4 min with pulses of 1 min. Then, the Petri dish was put in an ice water bath. The amylose and amylopectin content, functional properties, and microscopy analysis were determined in native and modified starches.

### 2.3. Amylose and Amylopectin Content of the Native and Modified Starch

Starch (100 mg), ethanol (1 mL, 96% *v*/*v*), and NaOH 1 M (9 mL) were placed in a volumetric flask, and the volume was adjusted with distillate water. An alíquot (5 mL) was transferred to a volumetric flask (100 mL) and mixed with acetic acid 1 M (1 mL) and iodine solution 2% (2 mL); then, the volume was adjusted with the distillate water [21]. The absorbance was measured in a UV/VIS spectrophotometer at 720 nm.

### 2.4. Functional Properties of Native and Physically Modified Starches

The following functional properties were determined in the native and modified starches: water absorption index, water solubility index, and swelling power according to Anderson et al. [22]. Starch samples (1.25 g) and water (30 mL) were incubated (60 °C, 30 min). Then, the samples were centrifuged (4900 rpm, 30 min). The supernatants (10 mL) were dried in an air oven (70 °C, 12 h) and weighed. The water absorption index (WAI) was expressed as the weight of gel obtained per gram of dry sample. The water solubility index (WSI) was expressed as the % of dry matter recovered after the supernatant was evaporated from the water sorption. The swelling power (SP) was measured as the weight of the swollen starch gel in relation to the dry weight of the starch.

The oil absorption capacity was evaluated according to Núñez-Breton et al. [20]. Vegetable oil (3 mL) was added to starch (0.5 g) and stirred (1 min); then, it was incubated (30 min, 24 °C). Afterwards, the sample was centrifuged, and the excess volume of oil was measured. The results were expressed as mL/g of retained oil.

### 2.5. Analysis of the Surface of Native and Modified Starches Using Microscopy Analysis

The surface of the native and modified starches was analyzed by using a force atomic microscopy (Multimode Veeco, Plainview, NY, USA) and Software Nanoscope 7.3 [23].

### 2.6. Formulation of Snacks

After the physical modification of starch and its analysis, the preparation of the snacks was performed. The snack was formulated using the following ingredients: amaranth flour, quinoa flour, nixmalizated corn (dough), native or modified starch of *Oxalis tuberosa*, onion powder, salt, and water (Table 1). The ingredients were mixed into a paste, which was left to rest (−20 °C, 20 min) and then placed into an electric pasta maker (Soda plus, mold pasta 2.5 mm). Then, the snacks were cooked at 180 °C for 6 min in an air fryer (T-FAL, 1450 W), and the cooking surface of the hot air frying machine was sprayed with commercial vegetable oil (0.09 g). The quality of the snacks was evaluated as described below.

### 2.7. Hardness and Radial Expansion Index

The physical properties, hardness and radial expansion index, of the snacks were analyzed as follows: hardness was evaluated using a TA-XT plus texture analyzer (Stable Micro Systems, Godalming, UK) [24]. The penetration rate, penetration distance, and spherical attachment for hardness tests were as follows: 1 mm/s, 3 mm, and 5 s/sp cm, respectively. Radial expansion index was determined using a vernier caliper to measure the snack diameter and expressed as the ratio of the snack diameter to the hole diameter of the pasta mold [25].

### 2.8. Hedonic Test

The sensory quality of snacks formulated with native and modified starches was evaluated using a bench-top test, with 30 judges and a hedonic test [26]. The acceptability, color, flavor, and texture of the snacks were evaluated with a scale of 7 points from “like much” to “dislike much”.

### 2.9. Colorimetric Parameters

To evaluate the color parameters L* a* b*, a colorimeter, Color Flex brand Hunter Lab model CX115 45/0, was used [27]. The hue angle (h°), chroma (C*), and browning index (BI) were calculated using the following equations:

h° = arctan (b*/a*)(1)

(2) $$C*=\sqrt{a^{2}+b^{2}}$$

BI = [100 ∗ (x − 0.31)]/0.172

where

x = (a + 1.75 L)/(5.645 L* + a* − 3.012 b*)(3)

### 2.10. Acrylamide Content

The analysis of acrylamide content was performed according to Biedermann et al. [28]. The sample (25 g) mixed with distillate water was put in an Erlenmeyer flask and heated in a water bath (70 °C, 30 min) under agitation. Isopropanol (40 mL) was added to the aliquot and centrifuged (3000× *g*, 10 min). Hexane was added to the supernatant and transferred to a separation funnel; then, the phase of interest, where the acrylamide remained, was placed in a flask, and anhydrous sodium carbonate was added. The analysis of acrylamide was performed in a gas chromatograph coupled to a mass spectrometer (Agilent Technologies 5975, Santa Clara, CA, USA) equipped with a carbowax column (30 m × 0.25 mm × 0.25 µm). The operation conditions were as follows: initial temperature of 80 °C (hold for 5 min) with an increase of 5 °C/min to 250 °C (15 min).

### 2.11. Proximal Composition Analysis

The proximate composition: moisture, ash, protein, and fat content were evaluated according to the AOAC [29].

### 2.12. Fatty Acid Profile by GC-MS

For the fatty acid analysis, lipids were previously extracted from the samples using hexane (1:5 *w*/*v*). Samples were placed in amber bottles and processed using an ultrasonic homogenizer (VCX 750, Sonics and Materials, Inc., Newtown, CT, USA, 20 kHz, 750 W) at 60% amplitude for 60 min with a 5 s on and 5 s off pulse. The procedure was performed twice. The organic phase was concentrated in a rotary evaporator to dryness and placed in an amber bottle under a nitrogen atmosphere and subsequently esterified with boron trifluoride [24]. The sample was injected into a gas chromatograph coupled to mass spectrometry (Agilent Technologies, model 5975) equipped with a DB-5 5% column (60 m × 250 µm nominal). The temperature program was as follows: 150 °C initial temperature held for 5 min with an increase of 5 °C/min to 225 °C. The carrier gas was helium (1 mL/min). Mass spectra were obtained by electron impact ionization (70 eV). Compound identification was performed with the database (HP Chemstation-NIST 05 Mass Spectral search program, version 2.0d), in addition to comparison with a standard (FAME mix, C8:C22, Cat. No. 18920-1AMP, Sigma-Aldrich) analyzed under the same conditions.

### 2.13. Statistical Analysis

For the data analysis, analysis of variance (ANOVA) and a Tukey mean comparison test were performed (*p* ≤ 0.05). XLSTAT 2024.3 was used to perform the statistical analyses.

## 3. Results and Discussion

### 3.1. Amylose/Amylopectin Content, Functional Properties, and Microscopy Analysis of Oxalis Tuberosa Starch

Table 2 shows that the amylose content of native starch (21.3%) is similar to previous data reported by Zhu and Cui [30]. Starch samples treated with liquid nitrogen and liquid nitrogen/microwaves had values in the range of 20.7–22.3%. A previous study showed a decrease in the amylose content of chemically modified starch of *Oxalis tuberosa* [20]. Furthermore, amylose from sorghum starch showed no significant change in content after physical modification by microwaves [31].

In addition, the amylopectin content as well as the amylose/amylopectin ratio of native starch and modified starch with liquid nitrogen and liquid nitrogen/microwaves are comparable to previous data reported by Fonseca-Santanilla and Betancourt-López [32]. It has been reported that freezing with liquid nitrogen can minimize structural damage to the starch [8,9,10]. In addition, a previous study indicates that potato starch modified with microwaves showed contents of amylose and amylopectin close to those found in native potato starch [17].

On the other hand, it has been reported that an increase in amylose is related to a higher water absorption [33]. In the present study, similar values of the water absorption index were observed for all starches, which is to be expected since the physical modification had no significant influence on the amylose content (Table 3).

On the other hand, swelling power is related to the amylose content and leached amylopectin; however, modified starches presented a similar swelling power to native starch (Table 3). Again, the physical modification of the starch of *Oxalis tuberosa* with liquid nitrogen and liquid nitrogen/microwaves did not alter the amylose and amylopectin content; therefore, the swelling power was not modified.

The water solubility index decreased significantly for starch treated with liquid nitrogen (11.6%) and with liquid nitrogen/microwaves (27.2%) as compared to native starch (Table 3). It has been reported that microwaves can lead to changes in the solubility of the starch [34] and reduce the amount of water needed in the snack formulation. [34,35].

The formulation of the snack with starch modified with liquid nitrogen and liquid nitrogen/microwaves required lower contents of water as compared to the snack formulated with native starch, especially the formulation with starch modified with liquid nitrogen/microwaves. It is well known that starches with a low solubility index require less water when preparing a product. This suggests that physical modification with liquid nitrogen/microwaves could reduce the water footprint of snack processing.

Regarding the oil absorption capacity, samples treated with liquid nitrogen and liquid nitrogen/microwaves showed 50% higher values than those shown by native starch (Table 2). Interestingly, previous studies reported that the chemically modified starch of *Oxalis tuberosa* presented a 210% higher oil absorption capacity as compared to native starch [20]. This suggests that physically modified starch with liquid nitrogen and microwaves has potential as a novel ingredient in the formulation of fried food by reducing the oil content of the final product.

**Figure 1:**
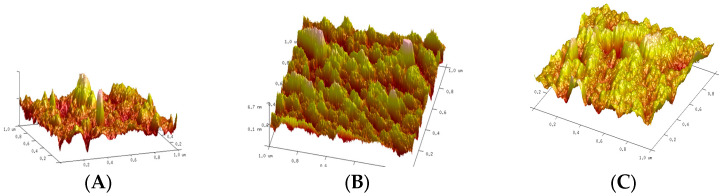
Pictures of atomic force microscopy of starch of *Oxalis tuberosa*: (**A**) native, (**B**) starch treated with liquid nitrogen, and (**C**) starch treated with liquid nitrogen/microwaves.

It has been reported that surface roughness also influences oil absorption in starchy foods. The Ra and Rq values, obtained from atomic force microscopy analysis, provide information on the impact of the modification on the surface roughness of the starch. The native starch, starch treated with liquid nitrogen, and starch treated with liquid nitrogen/microwaves showed roughness surface values (Ra) of 1.83, 1.5, and 2.48 nm, respectively (Figure 1). These data indicate a smooth surface for all starches. In addition, the values of mean square roughness (Rq) for native starch, starch treated with liquid nitrogen, and starch treated with liquid nitrogen/microwaves were 2.46, 1.87, and 3.71 nm, respectively. The Ra values reported for sweet potato starch are 1.05 nm and Rq 2.39 nm [36].

In addition, the surface roughness also influences the texture of starchy foods.

### 3.2. Texture and Radial Expansion Index of the Snacks

Texture is an important parameter in the quality of snacks. The hardness of the snack formulated with native starch was 3.06 ± 0.65 N, while the snacks formulated with starch treated with liquid nitrogen and liquid nitrogen/microwaves showed hardness values of 4.56 ± 1.04 and 4.83 ± 0.84 N, respectively, which were statistically different from the snack made of native starch. The snack containing starch treated with liquid nitrogen showed a higher value for hardness (49%) as compared to the snack of native starch. However, the snack formulated with liquid nitrogen/microwaves showed the highest hardness value (57%). It has been reported that cooked fresh noodles formulated with potato starch treated with microwaves showed the highest hardness values [15], and another study of puffed yellow pea snacks made by nitrogen-gas-assisted extrusion cooking showed an increase in hardness [16]. It is suggested that modified starch contributes to enhancing the texture of the snacks.

In addition, the snack formulated with native starch showed a radial expansion index of 1.16, while the snacks formulated with starch modified with liquid nitrogen and liquid nitrogen/microwaves showed diameter values of 1.36 and 1.19, respectively. It has been reported that the snack formulated with quinoa processed by extrusion cooking showed a decrease in the expansion [37].

### 3.3. Sensory Analysis

**Figure 2:**
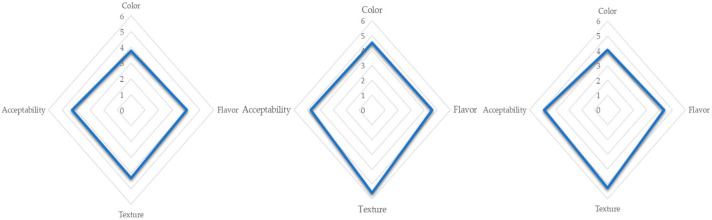
Acceptability, color, flavor, and texture of snacks formulated with native starch (SNS), starch treated with liquid nitrogen (SLNS), and starch treated with liquid nitrogen/microwaves (SLNMS).

A key factor in process innovation focused on low-fat foods is the sensory quality. Regarding hedonic tests, the snack formulated with starch treated with liquid nitrogen had the highest score for texture (5.6) as compared with the snack formulated with native starch (4.3) (Figure 2). The snack formulated with starch treated with liquid nitrogen/microwaves also showed a higher score for texture (5.3) than the snack formulated with native starch.

The flavor of the snack formulated with modified starch by liquid nitrogen (4.6) showed a higher score as compared to the snack formulated with native starch (4.0).

Besides texture, color is an important parameter in snacks, since a satisfactory color for consumers is a requirement of the industry. The hedonic test showed a higher score for color preference of snacks formulated with modified starch with liquid nitrogen (4.5) than snacks formulated with native starch (3.7) (Figure 2).

This suggests that the modified starch of *Oxalis tuberosa* is a novel option with a positive impact on texture, color, and flavor, which are important sensory parameters for the industry.

### 3.4. Color of the Snacks

Regarding the colorimeter parameters of snacks, the sample formulated with starch treated with liquid nitrogen showed the highest values of lightness (L*) as compared to that formulated with native starch, which indicated that modified starch contributes to the lightness of the snacks (Table 4). It is important to say that lightness (L*) is used for quality control in fried foods.

**Figure 3:**
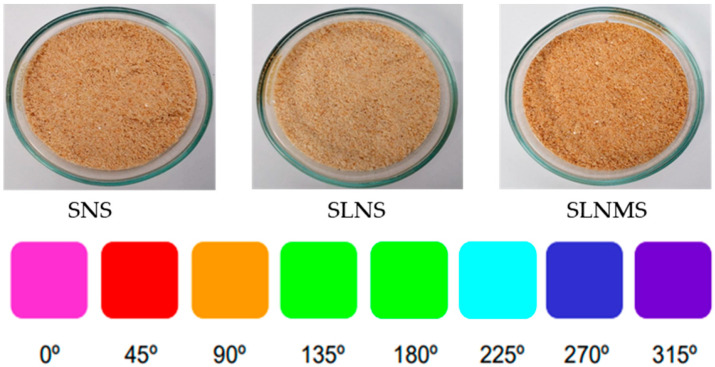
Color of snacks formulated with native starch (SNS), starch treated with liquid nitrogen (SLNS), and starch treated with liquid nitrogen/microwaves (SLNMS).

In addition, the snacks formulated with starch treated with liquid nitrogen showed the lowest value of the chroma C*, which indicates a less saturated color. Regarding the hue angle (Table 4), the snack formulated with starch treated with liquid nitrogen showed a higher value compared to the snack formulated with native starch, which had a slight reddish-yellow color (Figure 3).

On the other hand, the snack formulated with native starch showed a browning index of 77.4, while the snacks formulated with starch modified with liquid nitrogen and liquid nitrogen/microwaves presented a browning index of 43.9 and 54.8, respectively (Table 4). This data suggested that modified starch permits the obtaining of a snack with less browning. A previous study reported a lower BI value in cookies formulated with potato starch modified with microwaves, considering the L*, a*, and b* parameters and applying the browning index [17]. The BI has been used as an indicator of the development of the brown color in foods and has been associated with acrylamide content [38]. It has been reported that French fries processed by air hot frying showed the lowest browning index and acrylamide content as compared to French fries processed by deep frying and microwave frying [39].

### 3.5. Acrylamide Content

In the present study, the acrylamide content was not detected in snacks formulated with native and modified starch. It has been reported that the microwave treatment of starch can reduce the formation of acrylamide [40]. In addition, a previous study reported that previously freezing raw potatoes reduced the acrylamide content in chips and French fries, which was attributed to a decrease in the sugars and asparagine, precursors of acrylamide [41]. It is well known that acrylamide is formed during some cooking processes of foods, such as deep frying, especially in potatoes, due to the reaction between asparagine and reducing sugars. In addition, acrylamide can also be formed from acrolein, a compound mainly derived from the oxidation of lipids [3]. A previous study reported a reduction of 85–90% of acrylamide in potato products processed by hot air frying [42]. Among the factors that influence acrylamide formation is the moisture level of the food. A minimal acrylamide content was observed in a model system of starch matrices with water contents between 25 and 40%; at higher water contents, the acrylamide content was higher [43].

### 3.6. Proximal Composition and Fatty Acid Profile Analysis of the Snacks

Proximal composition analysis is a basic nutritional study of foods. The protein content of the snacks showed a value of 7.7 g/100 g of sample (Table 5). It indicates that the protein content was not reduced by hot air frying, which impacts the nutritional value of the snacks. This agrees with data reported for puffed snacks from Bengal gram and garbanzo grains processed by hot air frying [44].

In the present study, all snacks have a low-fat content (Table 5). It is noteworthy that the formulation of the snacks did not include vegetable oil, and it was only used to spray the cooking surface. So, the fat content comes mainly from the amaranth and quinoa used in the formulation. This suggests that hot air frying is a suitable method to obtain a low-fat plant-based snack.

In addition, the type and amount of dietary fat are important factors in the formulation of healthy snacks. The fatty acid profile of the snacks formulated with native and modified starch indicates that oleic and linoleic acids were the main components (Table 6). These data indicate that snacks provide ω-3, ω-6, and ω-9 fatty acids.

Given all the above, environmentally friendly technologies are an option to obtain modified starch from *Oxalis tuberosa*, which is a novel ingredient that can be used to develop healthy, low-fat, and plant-based snacks processed by hot air frying. This research provides information that can be used in the application of this sustainable processing on an industrial scale to obtain a desirable, nutritional, and safe low-fat and plant-based snack. Future work is necessary.

## 4. Conclusions

In the present study, the granules of starch from *Oxalis tuberosa* physically modified by liquid nitrogen and liquid nitrogen/microwaves showed changes in the water solubility index and oil absorption capacity. Starches modified via liquid nitrogen improved the texture and the color of the final products, which are important parameters from the sensory perspective. The snacks formulated with native starch and physically modified starch processed by hot air frying showed a low-fat content, mainly unsaturated fatty acids, and are also sources of protein. Acrylamide was not detected in any of the formulations. Environmentally friendly technologies are an option to obtain a modified starch of *Oxalis tuberosa*, which is a novel ingredient that can be used in healthy, low-fat, and plant-based snacks.

## Figures and Tables

**Table 1:** Formulation of snacks.

Ingredients (g/100 g)	SNS *	SLNS	SLNMS
Amaranth flour (g)	16.0	16.0	16.0
Quinoa flour (g)	16.0	16.0	16.0
Corn (g)	13.0	13.0	13.0
Native starch (g)	26.0	0.00	0.00
Starch treated with liquid nitrogen (g)	0.00	26.0	0.00
Starch treated with liquid nitrogen/microwaves (g)	0.00	0.00	26.0
Onion powder (g)	2.00	2.00	2.00
Salt (g)	2.00	2.00	2.00
Water (g)	25.0	23.6	21.1

* SNS = snack formulated with native starch; SLNS = snack formulated with starch treated with liquid nitrogen; SLNM = snack formulated with starch treated with liquid nitrogen/microwaves.

**Table 2:** Amylose and amylopectin content of native and physically modified starches from *Oxalis tuberosa*.

	Amylose (%)	Amylopectin (%)	Amylose/Amylopectin
NS	21.3 ± 0.04 ^a^	78.7 ± 0.04 ^b^	0.27 ± 0.00 ^c^
LNS	20.7 ± 0.00 ^a^	79.3 ± 0.00 ^b^	0.26 ± 0.00 ^c^
LNMS	22.3 ± 0.07 ^a^	77.7 ± 0.05 ^b^	0.28 ± 0.00 ^c^

NS = native starch, LNS = starch treated with liquid nitrogen, LNMS = starch treated with liquid nitrogen/microwaves. Different letters indicate statistically significant differences (*p* ≤ 0.05).

**Table 3:** Functional properties of native and modified starch of *Oxalis tuberosa*.

	* WAI (g Gel/g)	OAC (mL/g)	SP (%)	WSI (%)
NS ^✓^	2.23 ± 0.4 ^a^	1.2 ± 0.0 ^b^	2.33 ± 0.5 ^d^	4.19 ± 0.0 ^e^
LNS	2.25 ± 0.4 ^a^	1.8 ± 0.0 ^c^	2.34 ± 0.9 ^d^	3.7 ± 0.0 ^f^
LNMS	2.26 ± 0.9 ^a^	1.8 ± 0.0 ^c^	2.23 ± 0.7 ^d^	1.14 ± 0.2 ^g^

^✓^ NS = native starch, LNS = starch treated with liquid nitrogen, LNMS = starch treated with liquid nitrogen/microwaves. * WAI = water absorption index; OAC = oil absorption capacity; SP = swelling power; WSI = water solubility index. Different letters indicate statistically significant differences (*p* ≤ 0.05).

**Table 4:** L*, a*, b*, h°, C*, and browning index (BI) of the snacks.

Snacks	L*	a*	b*	h°	C*	BI
SNS	62.2 ± 0.2 ^a^	9.91 ± 0.1 ^c^	30.5 ± 0.3 ^f^	72.0 ±0.4 ^i^	32.1 ± 0.3 ^l^	77.4 ± 0.6 ^o^
SLNS	73.5 ± 0.3 ^a^	4.56 ± 0.2 ^d^	24.3 ± 0.4 ^g^	79.3 ± 0.7 ^j^	24.7 ± 0.4 ^m^	43.9 ± 0.5 ^p^
SLNMS	68.7 ± 0.0 ^b^	6.08 ± 0.0 ^e^	26.8 ± 0.1 ^h^	77.1 ± 0.0 ^k^	27.5 ± 0.1 ^n^	54.8 ± 0.2 ^q^

SNS = snack formulated with native starch; SLNS = snack formulated with starch treated with liquid nitrogen; SLNMS = snack formulated with starch treated with liquid nitrogen/microwaves. Different letters indicate statistically significant differences (*p* ≤ 0.05).

**Table 5:** Proximal analysis of snacks (g/100 g) formulated with native and physically modified starch of *Oxalis tuberosa*.

Snack *	Moisture	Ash	Protein	Fat	Carbohydrates
SNS	1.50 ± 0.15 ^b^	5.90 ± 1.98 ^c^	7.70 ± 0.4 ^f^	1.90 ± 1.22 ^g^	83.0 ± 3.37 ^i^
SLNS	1.60 ± 0.02 ^a^	6.00 ± 1.73 ^d^	7.70 ± 0.09 ^f^	2.70 ± 0.85 ^h^	82.0 ± 0.29 ^j^
SLMNS	1.65± 0.13 ^a^	7.11 ± 0.25 ^e^	7.75 ± 0.3 ^f^	1.49 ± 0.37 ^h^	82.0 ± 3.08 ^i^

* SNS = snack formulated with native starch; SLNS = snack formulated with starch treated with liquid nitrogen; SLNMS = snack formulated with starch treated with liquid nitrogen/microwaves. Data was expressed as g/100 g of sample. Different letters indicate statistically significant differences (*p* ≤ 0.05).

**Table 6:** Fatty acid profile of snacks made with native and physically modified starches of *Oxalis tuberosa*.

Fatty Acid (%)	Snacks *		
	SNS	SLNS	SLNSMS
C14:0 Myristic acid	0.04	0.05	0.08
C16:0 Palmitic acid	8.76	9.69	9.55
C18:0 Estearic acid	0.40	0.68	0.40
C16:1 Palmitoleic acid	0.07	0.09	0.07
C18:1 Oleic acid	45.3	38.2	39.9
C18:2 Linoleic acid	43.5	49.5	48.5
C18:3 Linolenic acid	1.93	1.79	1.50

* SNS = snack formulated with native starch; SLNS = snack formulated with starch treated with liquid nitrogen; SLNMS = snack formulated with starch treated with liquid nitrogen/microwaves.

## Data Availability

The datasets presented in this article are not readily available because the data are part of an ongoing study and to prevent commercial use of this research. Requests to access the datasets should be directed to the corresponding author.
